# Barriers and facilitators of tuberculosis treatment adherence among nomadic populations in Sub-Saharan Africa: A scoping review protocol

**DOI:** 10.1371/journal.pone.0340307

**Published:** 2026-01-13

**Authors:** Pablo Troop, Brenna Keam, Caroline Mullen, Julius Nyerere Odhiambo

**Affiliations:** 1 Ignite Global Health Research Lab, Global Research Institute, William and Mary, Williamsburg, Virginia, United States of America; 2 Department of Health Sciences, William and Mary, Williamsburg, Virginia, United States of America; Jaramogi Oginga Odinga University of Science and Technology, KENYA

## Abstract

**Introduction:**

Tuberculosis (TB) is a notable public health issue in sub-Saharan Africa (SSA), disproportionately affecting vulnerable populations such as nomads. Their migratory lifestyles limit consistent access to TB diagnostic and treatment services, leading to delays in diagnosis and challenges in completing the full TB treatment regimen. This scoping review aims to map the existing evidence on barriers and facilitators of TB treatment adherence among nomadic populations in SSA.

**Methods:**

The review will follow the Arksey and O’Malley methodological framework. Relevant published literature will be searched using a combination of keywords, Boolean terms, and Medical Subject Headings (MESH) across PubMed, Scopus, Google Scholar, and relevant grey literature sources. Eligible publications will include empirical research examining the barriers and facilitators that influence tuberculosis (TB) treatment adherence among nomadic populations. Study selection and data extraction will be undertaken independently by two reviewers, with discrepancies adjudicated by a third reviewer. The findings will be synthesized narratively in alignment with the Preferred Reporting Items for Systematic Reviews and Meta-Analysis Extension for Scoping Review (PRISMA-ScR) guidelines.

**Conclusion:**

This review seeks to map the existing evidence on the barriers and facilitators shaping TB treatment adherence among nomadic populations in sub-Saharan Africa. It is anticipated that this analysis will provide a foundation for a more targeted inquiry, support the development of context-appropriate TB strategies and guide policymakers in designing interventions better aligned with the needs of highly mobile populations.

**Trial registration:**

PROSPERO registration number CRD420250651525

## Introduction

Tuberculosis (TB), a communicable disease caused by *Mycobacterium tuberculosis,* is one of the leading infectious causes of mortality globally [1,2]. Although TB primarily affects the lungs, it can also manifest in extrapulmonary forms, involving multiple organ systems [1,3,4]. Despite being preventable and curable, TB causes significant morbidity and mortality, with over 10 million new cases and more than 1 million deaths annually, making it the leading cause of death from a single pathogen [5].

In 2014, the World Health Organization (WHO) launched the End TB Strategy, aiming for a 90% reduction in TB incidence between 2015 and 2035 [6]. However, global declines in TB incidence and mortality have been insufficient to meet these milestones or the Sustainable Development Goal (SDG) 3 target of ending the TB epidemic by 2030 [6,7]. Challenges in timely diagnosis, effective treatment, and comprehensive prevention—particularly in high-burden regions—continue to impede progress.

In Africa, progress has been uneven. While the region has achieved a 28% reduction in TB incidence and a 46% reduction in TB mortality since 2015, several countries still report some of the highest TB rates globally, exceeding 300 cases per 100,000 population in some areas and over 500 per 100,000 in Lesotho [5]. The situation is further exacerbated for countries with high levels of HIV co-infection—particularly in Southern Africa—where more than half of TB cases occur among people living with HIV. The region remains far from the 2025 End TB Strategy milestones of a 50% reduction in incidence and 75% reduction in mortality [5].

Despite generally high treatment success rates for drug-susceptible tuberculosis (TB), vulnerable populations face substantial barriers to diagnosis, treatment initiation, and adherence [8,9]. Nomadic pastoralist populations are particularly disadvantaged due to the structural constraints imposed by their mobile lifestyle, though the full extent of these challenges is not well understood. Previous studies have documented that seasonal movements in search of pasture and water disrupt continuity of care, delays diagnosis and treatment initiation and complicates adherence to prolonged TB regimens [10–14]. Limited access to WHO-recommended rapid diagnostic tests at peripheral health facilities, together with underreporting, leaves many cases undetected and untreated. Mobility not only increases the risk of treatment interruptions and disease progression but also sustains transmission within and between communities [15].

Treatment adherence is critical for tuberculosis (TB) outcomes, particularly for multidrug-resistant TB (MDR-TB), which requires regimens up to two years [16,17]. Among nomadic populations, adherence may be influenced by seasonal mobility, limited awareness of TB symptoms and transmission, and levels of community engagement. Socio-economic factors such as poorly ventilated shelters and geographically dispersed settlements further hinder access to care and continuity of treatment [13,14]. Yet, the relative contribution of these factors, and the ways in which they interact to influence treatment adherence in nomadic communities, remain poorly understood.

Stigma, particularly stemming from the perceived association between tuberculosis (TB) and HIV, emerges as another barrier. Fear of social judgment or being identified as having TB or HIV may discourage individuals from seeking diagnosis, initiating treatment, or adhering to prolonged therapy [12,18,19]. In nomadic populations, where healthcare engagement is already constrained by mobility and limited access to services, stigma may exert an even greater influence on health-seeking behavior. Poorly ventilated housing and dispersed settlements can reinforce social stigma, linking environmental risk factors with social perceptions of disease. The mechanisms through which stigma affects treatment decisions, and its magnitude across nomadic communities, remain unclear, representing a critical gap in understanding.

This scoping review protocol outlines a systematic approach to identify, map, and synthesize existing evidence on barriers and facilitators of TB treatment adherence among nomadic populations in sub-Saharan Africa. By focusing on socio-cultural, environmental, and structural determinants, the review aims to highlight knowledge gaps and inform context-specific interventions to improve adherence and treatment outcomes.

## Materials and methods

### Protocol design and registration

The review protocol has been registered on the International Prospective Register of Systematic Reviews database (http://www.crd.york.ac.uk/PROSPERO), registration number CRD420250651525.This scoping review will follow the Joanna Briggs Institute (JBI) manual for scoping reviews and adhere to the reporting standards outlined in the Preferred Reporting Items for Systematic reviews and Meta-Analyses extension for Scoping Reviews (PRISMA-ScR) [20,21]. The methodology will be guided by a six-staged framework proposed by Arksey and O’Malley [22], which includes: (1) identifying the research question, (2) identifying relevant studies, (3) study selection, (4) charting the data, (5) collating, summarizing, reporting results, and (6) consultation with tuberculosis experts. The optional sixth stage is conducted parallel with earlier stages [23].

### Stage 1: Identifying the research question

The primary question for the review is “What are the barriers and facilitators to tuberculosis (TB) treatment adherence among nomadic populations in Sub-Saharan Africa?”

The sub-questions are:

1. What are the key socio-cultural, environmental, and structural barriers to TB treatment adherence among nomadic populations in Sub-Saharan Africa?
2. How do stigma and discrimination associate with TB and HIV influence healthcare-seeking behavior and TB treatment adherence in nomadic communities?
3. What interventions or strategies have been effective in overcoming barriers to TB treatment adherence in nomadic populations, and what factors contribute to their success?

The study will employ the Population, Concept and Context framework, to determine the eligibility of the research questions (Table 1)

**Table 1:** Population, concept and context relating to the research question.

Criteria	Description
Population	Nomadic populations in Sub-Saharan Africa – Studies focusing on nomadic populations who are at risk for or affected by tuberculosis (TB), including individuals, families, and communities with limited or intermittent access to healthcare.
Concept	Studies examining the barriers and facilitators to TB treatment adherence, with an emphasis on socio-cultural, environmental, structural, and stigma-related factors that impact health-seeking behaviors and adherence to prescribed treatment protocols.
Context:	Nomadic living conditions in Sub-Saharan Africa, characterized by limited or inconsistent access to healthcare, inadequate housing conditions, and unique social structures. Studies included will focus on the impact of these contextual factors on TB care access and treatment adherence, as well as any interventions designed to address these challenges in nomadic communities. Time Frame – All publications from 2006 to the present will be considered. The year 2006 was chosen as the starting point due to the World Health Organization’s introduction of the End TB Strategy, which aimed to improve the availability and quality of TB data globally.

* SSA= Sub-Saharan Africa

### Stage 2: Identifying relevant studies

**Fig 1:**
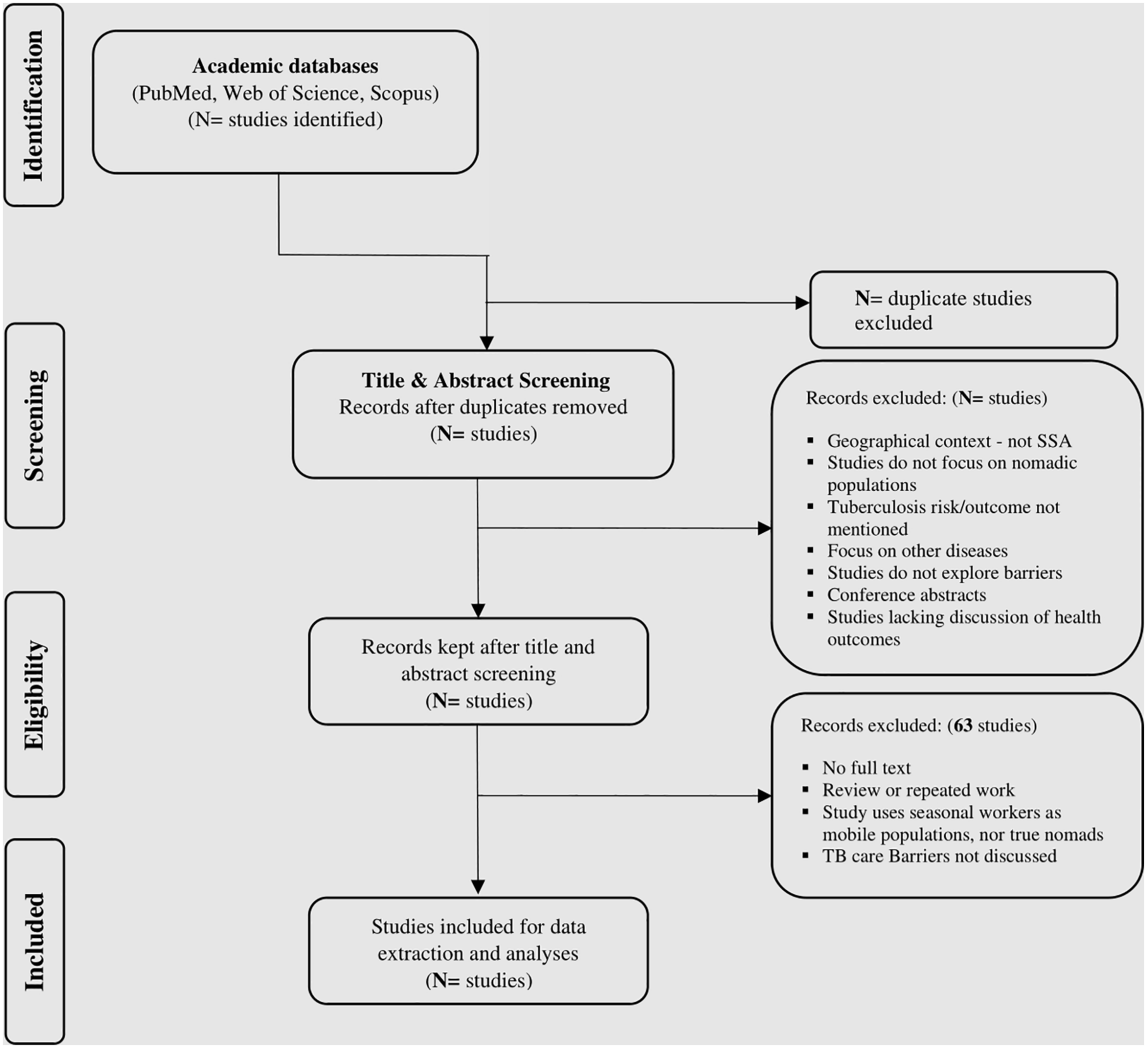
PRISMA-ScR flow diagram illustrating the selection of studies included in the scoping review.

Relevant studies on the barriers and facilitators of TB treatment adherence will be sourced from the following electronic databases: PubMed, Scopus, Google scholar The detailed search strategy is outlined in Search Strategy Fig 1a PRISMA-ScR flow diagram. The grey literature will also be reviewed for relevant studies. To ensure “in-depth and broad” retrieval of relevant literature [22] Additionally, reference lists of included studies will be manually reviewed to identify any further relevant literature.

### Eligibility criteria

Studies eligible for inclusion must meet the criteria outlined in Table 2.

**Table 2:** Inclusion and exclusion criteria.

Inclusion criteria	Exclusion criteria
Research examining barriers and facilitators to TB treatment adherence, including socio-cultural, environmental, structural, and stigma-related factors. Studies on TB care access and adherence in nomadic communities considering mobility, healthcare access and housing conditions Empirical studies (qualitative, quantitative, mixed-methods research and case studies). Studies published since 2006, aligning with the WHO End TB Strategy timeline. Studies will be limited to those published in English	Studies that focus on diseases other than TB Previous reviews Studies on non-nomadic populations or TB-affected groups outside Sub-Saharan Africa. Full text not accessible after reasonable retrieval attempts (institutional access, interlibrary loan, or author contact)

### Stage 3: Study selection

The selection of studies will be summarized using a PRISMA – ScR Study selection flow diagram (Fig 1). The researchers will pilot the search strategy to evaluate its relevance to the selected electronic databases and keywords for the study. To enhance the search comprehensiveness, thematic search terms will be combined using Boolean operators, and Medical Subject Headings (MeSH) will be applied and adjusted as needed for each database (S1 Table). The principal investigator will oversee title screening, while two independent reviewers will conduct abstract screening. Full-text screening will also be done independently based on the eligibility criteria. In cases of disagreement between the reviewers, a third reviewer will be used if any disagreements on study selection occur. Furthermore, any disagreements will first lead to a discussion between the two reviewers, during which each reviewer will explain their rationale for inclusion or exclusion based on the predefined eligibility criteria. If the reviewers are unable to reach consensus after discussion, the disagreement will be referred to a third reviewer, who will make the final inclusion decision. All excluded articles at the full-text stage will be recorded in a “Reasons for Exclusion” spreadsheet that documents the specific justification for exclusion for each study. If an article is not freely accessible online, assistance will be sought from the institution’s library services. Additionally, the original authors may be contacted via email to request full-text access if necessary. Cohen’s kappa coefficient (κ) will be calculated to assess inter-rater agreement between reviewers at the conclusion of the full-text screening phase.

### Quality appraisal

To assess the quality of the studies, the Mixed Method Appraisal Tool (MMAT) version 2020 will be used [24]. This tool will evaluate the study’s aim, methodology, design, participant recruitment, data collection and analysis processes, findings, discussions, and conclusions. Additionally, the authors will cross-examine the identified themes and assess their relevance to the research question, considering the findings’ meanings and their implications for future research. While it is not typical to include quality appraisal in a systematic review, the authors have decided to include quality appraisal to enhance the interpretability and usefulness of our findings.

### Stage 4: Charting the data

Two independent reviewers will extract relevant data from the full-text articles after thorough reading. A hybrid approach, combining both inductive and deductive methods, will guide the data extraction process. The form as shown in S2 Table will capture the following details: (1) author and year of publication, (2) title of study, (3) aims and objectives, (4) country of study, (5) study design, (6) study participants, (7) study results, (8) findings related to the research question, (9) conclusion, and (10) recommendations. The form will be continuously updated to ensure all relevant data are captured to address the review question effectively. If any information is missing then it will be cataloged as not stated under the appropriate column in the data extraction form.

### Stage 5: Collating, summarizing and reporting the results

We will employ thematic content analysis to identify and describe patterns relevant to the research question. This process will begin with initial coding and categorization of extracted qualitative data, followed by iterative refinement to develop key themes. Specifically, we will focus on themes related to barriers and facilitators of TB treatment adherence among nomadic populations in Sub-Saharan Africa. Coding will be conducted independently by multiple reviewers, and discrepancies will be resolved through discussion to ensure rigor and consistency.

Themes will be organized into higher-level categories that reflect structural, cultural, and health-system factors influencing adherence. Findings will be presented using summary tables, thematic maps, and narrative synthesis, highlighting relationships between themes and their contextual relevance. Where possible, illustrative quotes from primary studies will be included to enrich interpretation.

In line with scoping review methodology, our synthesis will aim to map the breadth and nature of evidence rather than assess effect sizes. However, if sufficient quantitative data are available, we will consider presenting descriptive statistics (e.g., frequencies, proportions) to summarize the distribution of barriers and facilitators across contexts. This may include tables and charts showing the prevalence of specific themes by country, population subgroup, or intervention type.

### Stage 6: Consultation

An experienced TB researcher will provide expert guidance throughout all the stages of the review, including in the planning, execution, and dissemination phases.

### Ethics statement

This scoping review will be conducted using previously published literature and does not involve human or animal participants. Ethical approval is not required.

## Discussion

Adherence to tuberculosis (TB) treatment is a critical determinant of treatment success, yet suboptimal adherence remains a significant barrier to the achievement of TB elimination targets. A comprehensive understanding of the factors influencing adherence is essential for optimizing treatment outcomes and advancing the global agenda of TB eradication. This scoping review will be the first to systematically synthesize evidence on the barriers and facilitators of TB treatment adherence among nomadic populations in Sub-Saharan Africa (SSA), a demographic characterized by unique challenges, including inconsistent access to healthcare, stigma, and mobility-related disruptions to care. Although this review will be limited to studies published in English, potentially introducing language bias, it will provide a nuanced understanding of the adherence dynamics specific to this vulnerable population. The findings will be instrumental in identifying key intervention strategies, such as mobile healthcare units, community-based support networks, and culturally tailored educational campaigns. By aligning with the World Health Organization’s End TB Strategy, which targets the elimination of TB by 2035 through a comprehensive, patient-centered approach, this review will contribute to evidence-based recommendations for improving treatment adherence and advancing TB control efforts in SSA’s most marginalized communities.

## Supporting information

S1 Table Search Strategy. (DOCX)

S2 Table Data Extraction Form. (DOCX)
