# Supplementary material for: Barriers and facilitators of tuberculosis treatment adherence among nomadic populations in Sub-Saharan Africa: A scoping review protocol
Source: PLoS One. 2026 Jan 13;21(1):e0340307. doi: 10.1371/journal.pone.0340307 (PMC12798961; doi:10.1371/journal.pone.0340307)
Supplement: S1 Table — (DOCX) [file pone.0340307.s001.docx]

#### **Supplementary Table 1: Search strings**

| Database | Date of search | Keywords | # of publications retrieved |
| --- | --- | --- | --- |
| PubMed | November 7, 2025 | ("tuberculosis" OR "TB") AND ("adherence" OR "compliance" OR "treatment default" OR "treatment dropout" OR "treatment interruption" OR "loss to follow-up" OR "treatment barriers" OR "access to care" OR "access to treatment" OR "healthcare access") AND ("pastoralist" OR "nomadic" OR "transhumant" OR "semi-nomadic" OR "mobile population" OR "mobile community" OR "migrant population" OR "displaced persons" OR "hard-to-reach population" OR "remote community" OR "cross-border population" OR "rural population" OR "pastoral community" OR "pastoral society" OR "agropastoralist" OR "pastoral nomads") AND ("Africa" OR "Sub-Saharan Africa" OR "Sahel" OR "Horn of Africa" OR "Ethiopia" OR "Kenya" OR "Somalia" OR "Chad" OR "Sudan" OR "South Sudan" OR "Niger" OR "Mali" OR "Nigeria" OR "nomadic tribe" OR "nomadic group" OR "pastoral region") NOT ("cattle" OR "livestock" OR "animal health" OR "veterinary" OR "zoonotic") | 72 |
| Google Scholar | November 7, 2025 | "tuberculosis treatment" AND ("barriers" OR "facilitators" OR "challenges" OR "enablers" OR "access to care" OR "health service delivery") AND ("nomadic populations" OR "pastoralist communities" OR Fulani OR Maasai OR "Somali pastoralists" OR "mobile populations" OR "transhumant groups") AND ("Sub-Saharan Africa" OR Nigeria OR Kenya OR Ethiopia OR Uganda OR Sahel OR "East Africa") | 422 |
| Scopus | November 7, 2025 | TITLE-ABS-KEY (tuberculosis OR TB) AND TITLE-ABS-KEY (nomad* OR pastoralist* OR "mobile population" OR migrant*) AND TITLE-ABS-KEY ("Sub-Saharan Africa" OR Africa OR Kenya OR Ethiopia OR Nigeria OR Uganda OR Sahel) AND NOT TITLE-ABS-KEY (Sweden OR Europe OR Italy OR France OR Spain) | 282 |
