# Supplementary material for: Barriers and facilitators of tuberculosis treatment adherence among nomadic populations in Sub-Saharan Africa: A scoping review protocol
Source: PLoS One. 2026 Jan 13;21(1):e0340307. doi: 10.1371/journal.pone.0340307 (PMC12798961; doi:10.1371/journal.pone.0340307)
Supplement: S2 Table — (DOCX) [file pone.0340307.s002.docx]

Supplementary Table 2- Data extraction form

| Category | Details |
| --- | --- |
| Bibliographic Information | Study ID  • Author(s), Year  • Country/Region (Specify if study focuses on Sub-Saharan Africa)  • Article Title  • Language  • Study Period (Start-End Years)  • Data Source (National TB programs, medical records, surveys, mobile clinics, others)  • Type of Publication (Journal article, book chapter, grey literature, government reports, WHO reports)  • Study Population (Nomadic groups, pastoralists) |
| Aims | Study aims and objectives |
| Methodology | • Data Sources Used (Surveys, interviews, health records)  • Multiple Sources Used? (Yes/No - List if applicable)  • Study Design (Qualitative, cross-sectional etc)  • Sampling (How nomadic populations were identified and studied) |
| Results | • Key Findings (Prevalence rates, transmission patterns, high-risk locations, mobile populations, identified structural and cultural barreirs)  • Unexpected Results (Findings that contradict previous knowledge) |
| Discussion | • Key Findings  • Unexpected Results  • Gaps in TB Surveillance & Research on nomadic roups |
| Conclusions and recommendations | • Suggested Improvements for Future TB Surveillance & Research in nomadic Populations Better data integration from multiple sources  • Need for cross-border TB surveillance for migratory groups  • Barriers or gaps requiring further research • Use of mobile health units |
